# Bortezomib Is Cytotoxic to the Human Growth Plate and Permanently Impairs Bone Growth in Young Mice

**DOI:** 10.1371/journal.pone.0050523

**Published:** 2012-11-30

**Authors:** Emma Eriksson, Farasat Zaman, Dionisios Chrysis, Henrik Wehtje, Terhi J. Heino, Lars Sävendahl

**Affiliations:** 1 Pediatric Endocrinology Unit, Department of Women’s and Childreńs Health, Karolinska Institutet, Stockholm, Sweden; 2 Pediatric Endocrinology Unit, Department of Pediatrics, University of Patras, Rio, Greece; 3 Pediatric Orthopedic Clinic, Department of Women’s and Children's Health, Karolinska Institutet, Stockholm, Sweden; 4 Department of Anatomy, Institute of Biomedicine, University of Turku, Turku, Finland; Ohio State University, United States of America

## Abstract

Bortezomib, a novel proteasome inhibitor approved for the treatment of cancer in adults, has recently been introduced in pediatric clinical trials. Any tissue-specific side effects on bone development have to our knowledge not yet been explored. To address this, we experimentally studied the effects of bortezomib *in vivo* in young mice and *in vitro* in organ cultures of rat metatarsal bones and human growth plate cartilage, as well as in a rat chondrocytic cell line. We found that bortezomib while efficiently blocking the ubiquitin/proteasome system (UPS) caused significant growth impairment in mice, by increasing resting/stem-like chondrocyte apoptosis. Our data support a local action of bortezomib, directly targeting growth plate chondrocytes leading to decreased bone growth since no suppression of serum levels of insulin-like growth factor-I (IGF-I) was observed. A local effect of bortezomib was confirmed in cultured rat metatarsal bones where bortezomib efficiently caused growth retardation in a dose dependent and irreversible manner, an effect linked to increased chondrocyte apoptosis, mainly of resting/stem-like chondrocytes. The cytotoxicity of bortezomib was also evaluated in a unique model of cultured human growth plate cartilage, which was found to be highly sensitive to bortezomib. Mechanistic studies of apoptotic pathways indicated that bortezomib induced activation of p53 and Bax, as well as cleavage of caspases and poly-ADP-ribose polymerase (PARP) in exposed chondrocytes. Our observations, confirmed *in vivo* and *in vitro*, suggest that bone growth could potentially be suppressed in children treated with bortezomib. We therefore propose that longitudinal bone growth should be closely monitored in ongoing clinical pediatric trials of this promising anti-cancer drug.

## Introduction

Longitudinal bone growth takes place within the epiphyseal growth plate of long bones by a process called endochondral ossification, which is the result of the creation of bone tissue from a cartilage scaffold. Apoptosis is necessary for controlling cell number to maintain homeostasis and normal function in most organs, including the growth plate [1], [2], [3]. The targeting of apoptosis is also a key mechanism of cancer therapeutics. Survivors of pediatric cancers often display severe long-term complications secondary to previously administered life-saving treatment. Consequently, due to the increased numbers of childhood cancer survivors, the long-term effects of cancer treatment have attracted recent interest. Previous reports have suggested that anti-cancer therapeutics used to treat childhood malignancies act directly on epiphyseal growth plate chondrocytes, resulting in reduced adult height of surviving children [4], [5], [6], [7], [8]. Furthermore, previous data suggest that the growth plate is damaged in children with acute lymphoblastic leukemia who have received sustained and intensive chemotherapy [9]. Remarkably, despite the existing clinical evidence, there have been few investigations into the direct effects of cytotoxic chemotherapy on skeletal growth and growth plate cartilage.

Proteasome inhibitors constitute a novel class of antitumor agents with preclinical evidence of activity against hematologic malignancies and solid tumors [10]. Specifically, bortezomib, a boronic acid dipeptide with potent and selective effects, acts as an inhibitor of the 26S proteasome [11], and is the first-in-class proteasome inhibitor approved for the treatment of relapsed/refractory multiple myeloma and mantle cell lymphoma in adults [12], [13]. The important biological effects of bortezomib include the induction of apoptosis and initial accumulation of the tumor suppressor protein, p53 [14], which can promote apoptosis by several mechanisms [15]. Bcl-2 family members have been shown to be p53 targets; the pro-apoptotic member, Bax, is up-regulated in a number of systems during p53-mediated apoptosis whereas the anti-apoptotic Bcl-2 family member, Bcl-2, is down-regulated [16].

Although we and others have experimentally demonstrated that non-clinically used proteasome inhibitors can induce chondrocyte apoptosis [17], [18], [19], the effect on bone growth and the underlying mechanism by which the clinically used proteasome inhibitor bortezomib affects growth plate chondrocytes remains to be elucidated. Bortezomib appears to be more effective as an anti-cancer drug than other proteasome inhibitors and has therefore rapidly entered into clinical practice, and now also into clinical trials in childhood cancers [20], [21]. Although the increased efficacy is important for the treatment of pediatric cancers, it should be kept in mind that the increased potency might also affect normal tissues and organs in growing individuals. The present experimental study was designed to address whether bortezomib treatment affects longitudinal bone growth and bone homeostasis, and if so, what the underlying cellular mechanisms are. Studies were performed both *in vivo* and *in vitro* using two strains of young mice, cultured metatarsal bones and chondrocytes of rat origin. Finally, we also used tissue specimens of human growth plate cartilage to assess the toxicity of bortezomib.

## Results

### Bortezomib Inhibits the UPS in Treated Mice

To verify that bortezomib caused efficient proteasome inhibition, proteasomal activity was assessed in whole blood collected at 0, 1, 24 and 72 hrs after one systemic injection of either bortezomib (1 mg/kg) or vehicle. One hour after bortezomib injection, proteasome activity was decreased by 74±0.4% and 53±3.1% in C57B and NMRI mice, respectively (p<0.001 vs. vehicle for both strains). These data indicate a similar degree of proteasome inhibition as seen in treated humans, i.e. within the 50–80% range [22]. When assessed 72 hrs after the injection with bortezomib, proteasome activity had almost fully recovered to baseline level (data not shown).

### Bortezomib Induces Chondrocyte Toxicity and Bone Growth Impairment in Treated Mice

**Figure 1:**
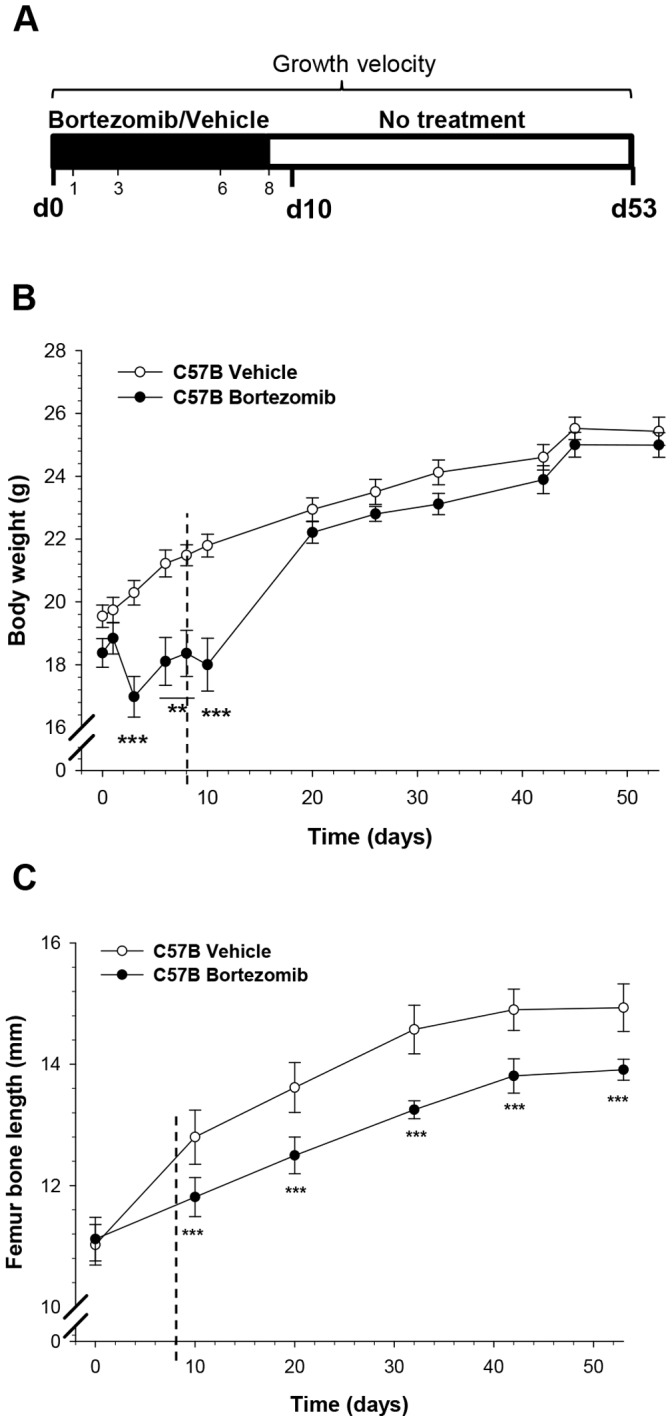
Permanent growth impairment in bortezomib-treated C57B mice fed *ad libitum.* A) Schematic illustration of bortezomib treatment in C57B mice. Mice fed *ad libitum* received 1 cycle of i.p. injected bortezomib (1 mg/kg) on day 1, 3, 6 and 8, or vehicle, and then were sacrificed on day 10 (d10, 48 hrs post-treatment) or day 53 (d53, 45 days post-treatment). **B)** Mean body weight throughout the experiment. Between day 3 and 10, a significant decrease in body weight of bortezomib treated mice was observed compared to vehicle (**p<0.01, ***p<0.001). **C)** Permanent bone growth impairment was observed at day 10 (48 hrs after last injection) and onwards, with significant decrease in femur length of bortezomib treated mice vs. vehicle (***p<0.001). The vertical dashed lines in panels B and C indicate the last injection of bortezomib or vehicle. Data represent mean±SEM.

To verify if inhibition of the UPS has any impact on linear bone growth, C57B mice were fed *ad libitum* and treated with one cycle of bortezomib or vehicle as indicated in Fig. 1A. Already when assessed on day 10 (48 hrs after last injection), bone growth was clearly decreased in bortezomib treated animals (Fig. 1C). The total femur growth from day 0 to 10 was 0.34±0.20 mm and 1.64±0.12 mm in bortezomib and vehicle treated animals, respectively (p<0.001). Body weight decreased during bortezomib treatment (p<0.001 vs. vehicle; Fig. 1B), whereas food intake was less affected (6.62±1.49 g/day for vehicle vs. 4.93±1.17 g/day for bortezomib, not statistically significant). After the treatment period with bortezomib, there was a quick catch-up in body weight and at the end of the study (d53) no difference existed between the groups (Fig. 1B). In contrast, bone growth did not catch-up and consequently also on day 53 bone length was shorter in the bortezomib group (p<0.001 vs. vehicle; Fig. 1C).

### Bortezomib Induces Permanent Bone Growth Impairment in Pair-fed Mice

**Figure 2:**
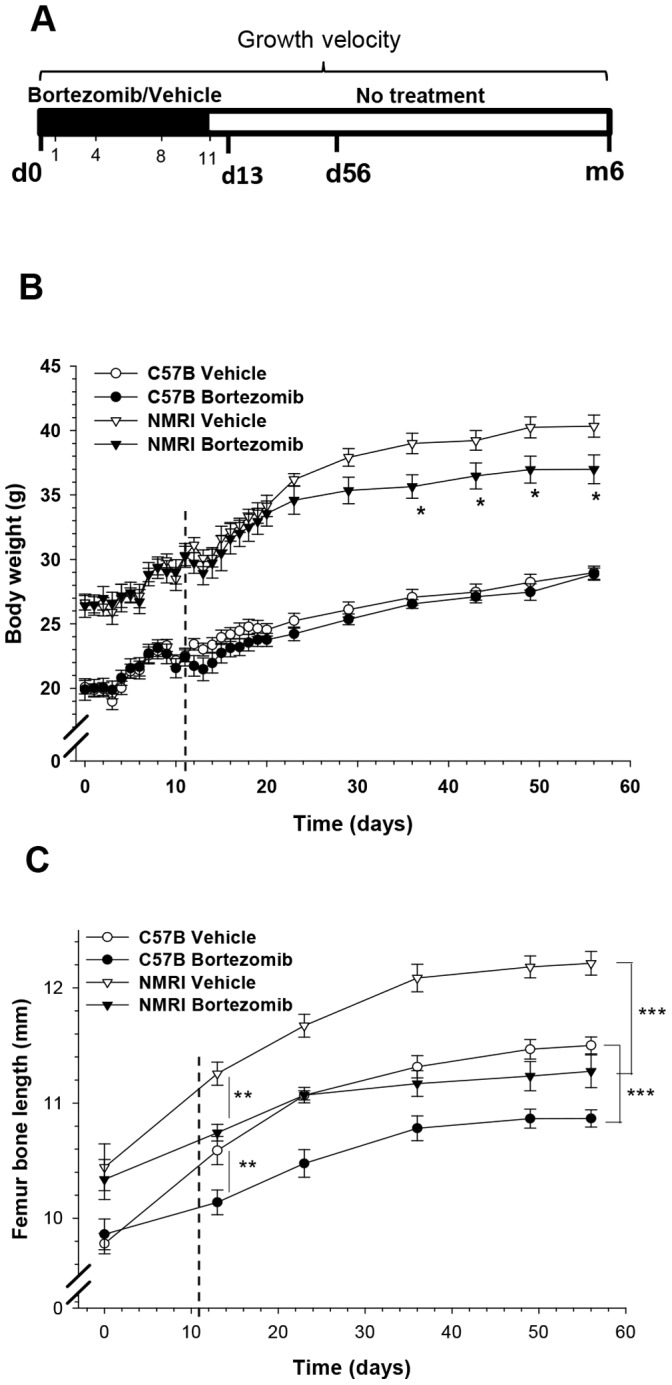
Bortezomib treatment caused permanent bone growth impairment in pair-fed animals. A) Schematic illustration of bortezomib treatment in C57B and NMRI mice. At start of treatment, day 0 (d0), mice received either 1 cycle of i.p. injected bortezomib (1 mg/kg) or vehicle on day 1, 4, 8 and 11, and were then sacrificed on day 13 (d13, 48 hrs post-treatment), day 56 (d56, 45 days post-treatment) or after 6 months (6 m). **B)** Animals were pair-fed and body weight was measured every day until day 20 after start of treatment, thereafter food was provided *ad lib.,* and body weight was measured once per week. From day 36 onwards body weight increase was suppressed in NMRI treated mice (*p<0.05 vs. vehicle). **C)** Permanent bone growth impairment was observed from day 13 until termination on day 56, with a significant decrease in femur length of bortezomib treated mice vs. vehicle of the same strain (**p<0.01 on d13 and ***p<0.001 on d56). The vertical dashed lines in panels B and C indicate the last injection of bortezomib. Data represent mean±SEM.

To study if the observed growth impairment caused by bortezomib could be secondary to a nutritional effect, two different strains of mice (n = 8–10 per group) were weight-matched, pair-fed and treated with 1 cycle of bortezomib (Fig. 2A) which did not affect body weight during the treatment period although later on a slower weight increase was noted in the NMRI strain (Fig. 2B). Pair-fed mice of both these strains demonstrated clear bone growth impairment when treated with bortezomib (Fig. 2C). Femur bone growth between day 0 to 13 was 0.62±0.11 mm in bortezomib and 1.37±0.17 mm in vehicle treated C57B mice (p<0.01) while in NMRI mice bone growth was 0.75±0.15 mm and 1.63±0.11 mm, respectively (p<0.001). Still on day 56 (45 days post-treatment), femur bone length was significantly shorter in bortezomib treated mice of both strains (p<0.001 vs. vehicle; Fig. 2C) confirming a lack of catch-up growth. When C57B animals were followed for 6 months after the last injection femur length was still shorter in previously bortezomib treated mice when compared to vehicle (14.9±0.0 mm vs. 15.3±0.1 mm; p<0.01) while no differences in body weight was observed (data not shown).

### Bortezomib Acts Locally in the Growth Plate to Inhibit Linear Bone Growth

**Figure 3:**
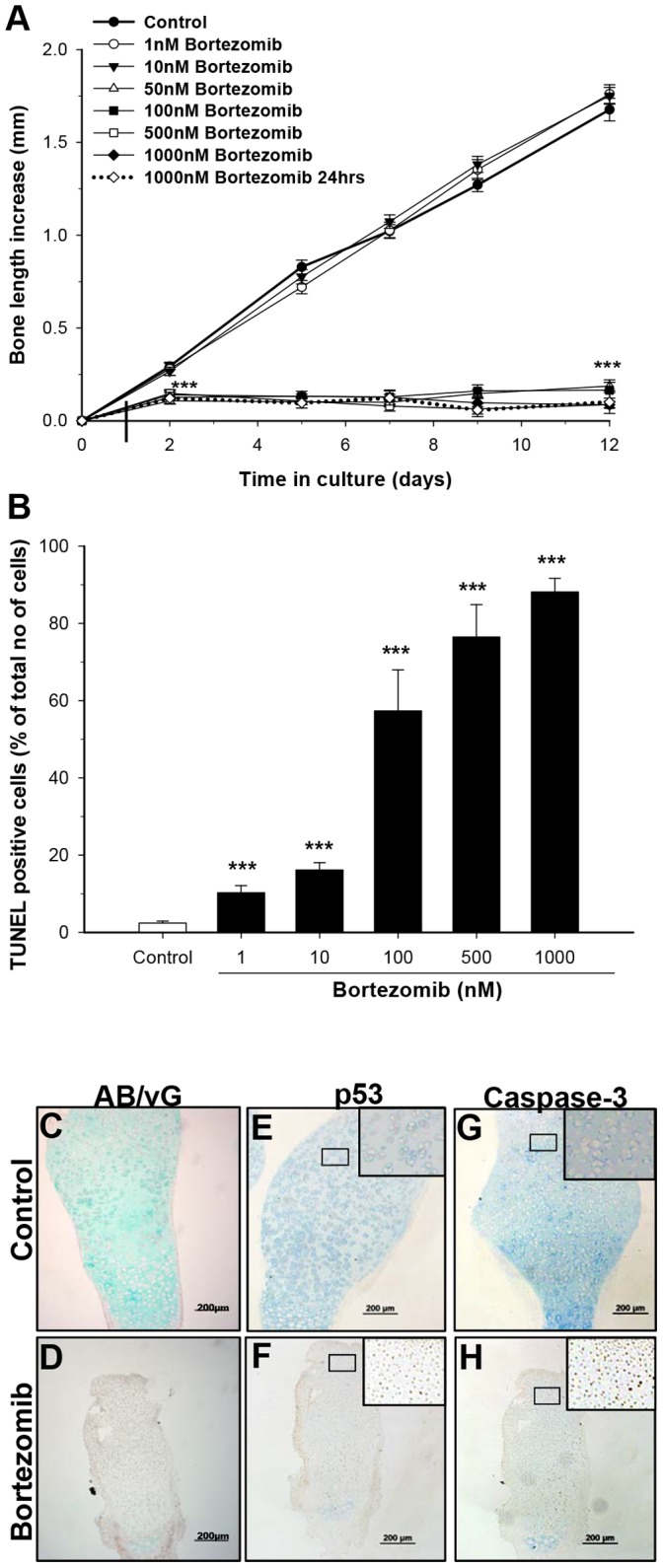
Bortezomib locally effects chondrogenesis, leading to growth impairment in *ex vivo* cultured rat metatarsal bones. **A)** Fetal (E20) rat metatarsal bones were dissected out and cultured *ex vivo* with or without bortezomib for 12 days (1–1000 nM). Bortezomib, at concentrations above 50 nM caused sustained bone growth impairment (***p<0.001 vs. control), whereas concentrations of 1 nM and 10 nM did not have an effect. For one of the groups, bortezomib (1000 nM) treatment was restricted to the first 24 hrs in culture (vertical line), and thereafter bones were transferred to control medium (without bortezomib; ***p<0.001 vs. control). **B)** Metatarsals were TUNEL stained for apoptosis (***p<0.001 vs. control). **C, D)** Alcian Blue/van Gieson (AB/vG) staining of E20 metatarsals showed significant matrix deposition (blue color) in bones cultured for 12 days in control medium (**C**) while bones cultured with bortezomib (1000 nM) showed minimal deposition of matrix (**D**). Immunohistochemical staining for p53 (**E, F**) and active caspase-3 (**G, H**) showing increased expression of both pro-apoptotic proteins after bortezomib treatment (1000 nM; **F** and **H**, respectively) compared to control bones (**E** and **G**, respectively) when analyzed. Data represent mean±SEM.

To rule out if the observed bone growth impairment was mediated through systemic modulation of the GH/IGF-I axis, analyses of serum IGF-I levels were performed. Bortezomib treatment did not affect serum IGF-I in C57B mice (279.3±19.1 ng/ml vs. 282.4±16.7 ng/ml in vehicle). While in NMRI mice, there was a significant increase in serum IGF-I levels when assessed on day 13 of bortezomib treatment (470.8±22.5 ng/ml vs. 342.3±20.0 ng/ml in vehicle; p<0.001). On day 56 there was no difference in IGF-I levels between bortezomib and vehicle treated mice in any of the strains (data not shown). These minimal effects of bortezomib treatment on serum IGF-I levels indicate a more local effect of bortezomib in the growth plate. To further investigate this, fetal (E20) rat metatarsal bones were cultured for 12 days in the presence of bortezomib (1–1000 nM; Fig. 3A). Significant bone growth impairment was observed as early as 2 days after exposure to bortezomib at 50 nM or higher concentrations (Fig. 3A; p<0.001 50 nM bortezomib vs. control). Interestingly, when exposed for only 24 hrs to 1000 nM bortezomib, a permanent growth arrest was still induced (Fig. 3A, dotted line; p<0.001 vs. control). A similar permanent bone growth retardation was found when postnatal (P8) metatarsal bones were cultured with 100, 500 and 1000 nM bortezomib for 12 days (p<0.001 for all concentrations tested vs. control on day 12, data not shown). Taken together, our data support a local effect of bortezomib in the growth plate with irreversible chondrocyte damage.

### Bortezomib Impairs Chondrogenesis and Induces Chondrocyte Apoptosis

**Figure 4:**
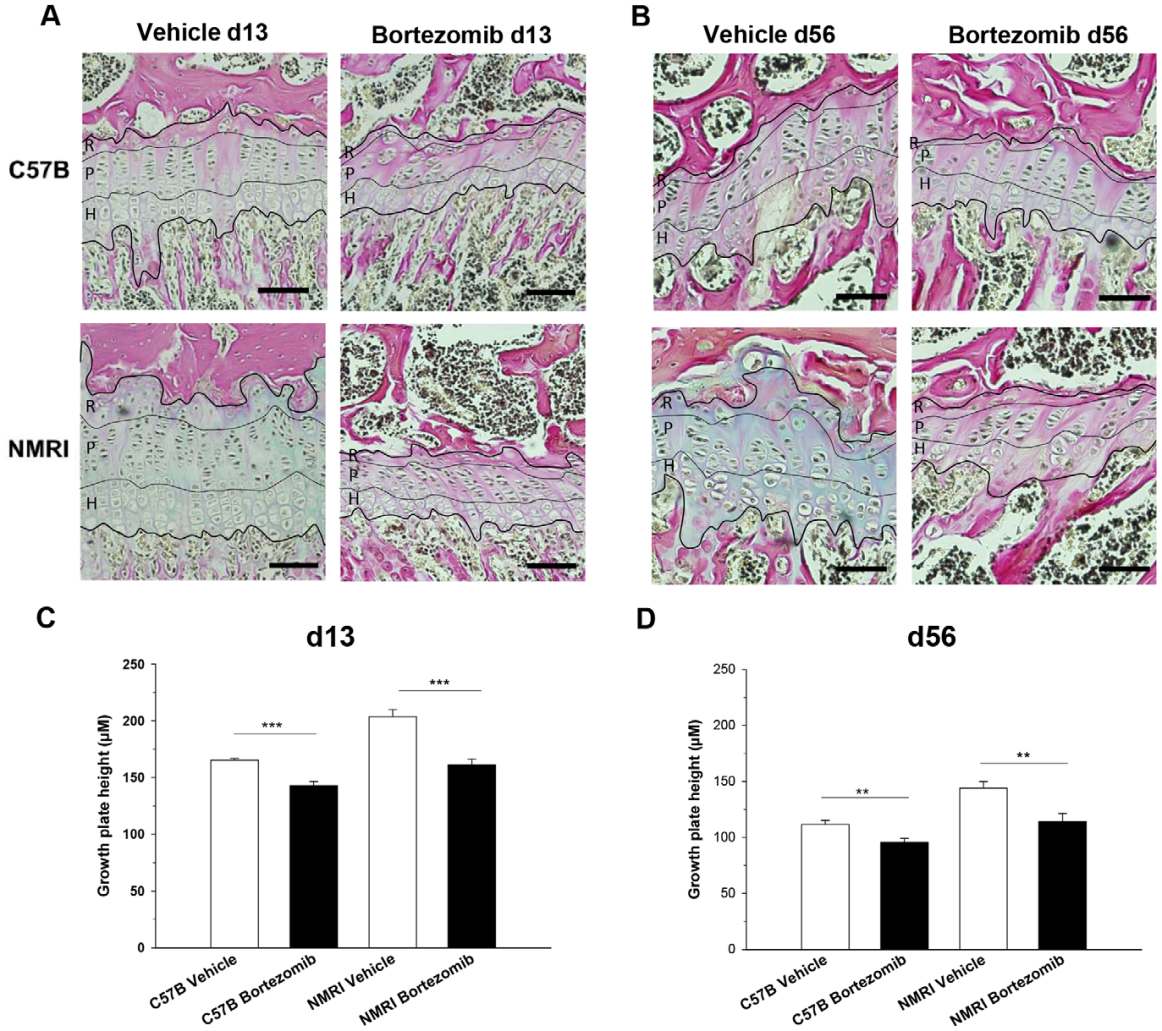
Bortezomib significantly reduced growth plate height. Representative pictures of mouse tibia growth plates on day 13 (**A**) and at termination of the experiment on day 56 (**B**). The thin black lines indicate the different zones within the growth plate; R (resting), P (proliferative) and H (hypertrophic). Bars, 100 µm. Quantitative analyses of growth plate height on day 13 (**C**) and on day 56 (**D**) within the tibia growth plates of vehicle- and bortezomib treated C57B and NMRI mice, n = 6 per group. Data represent mean±SEM; **p<0.01, ***p<0.001 vs. corresponding strain of vehicle treated mice.

**Figure 5:**
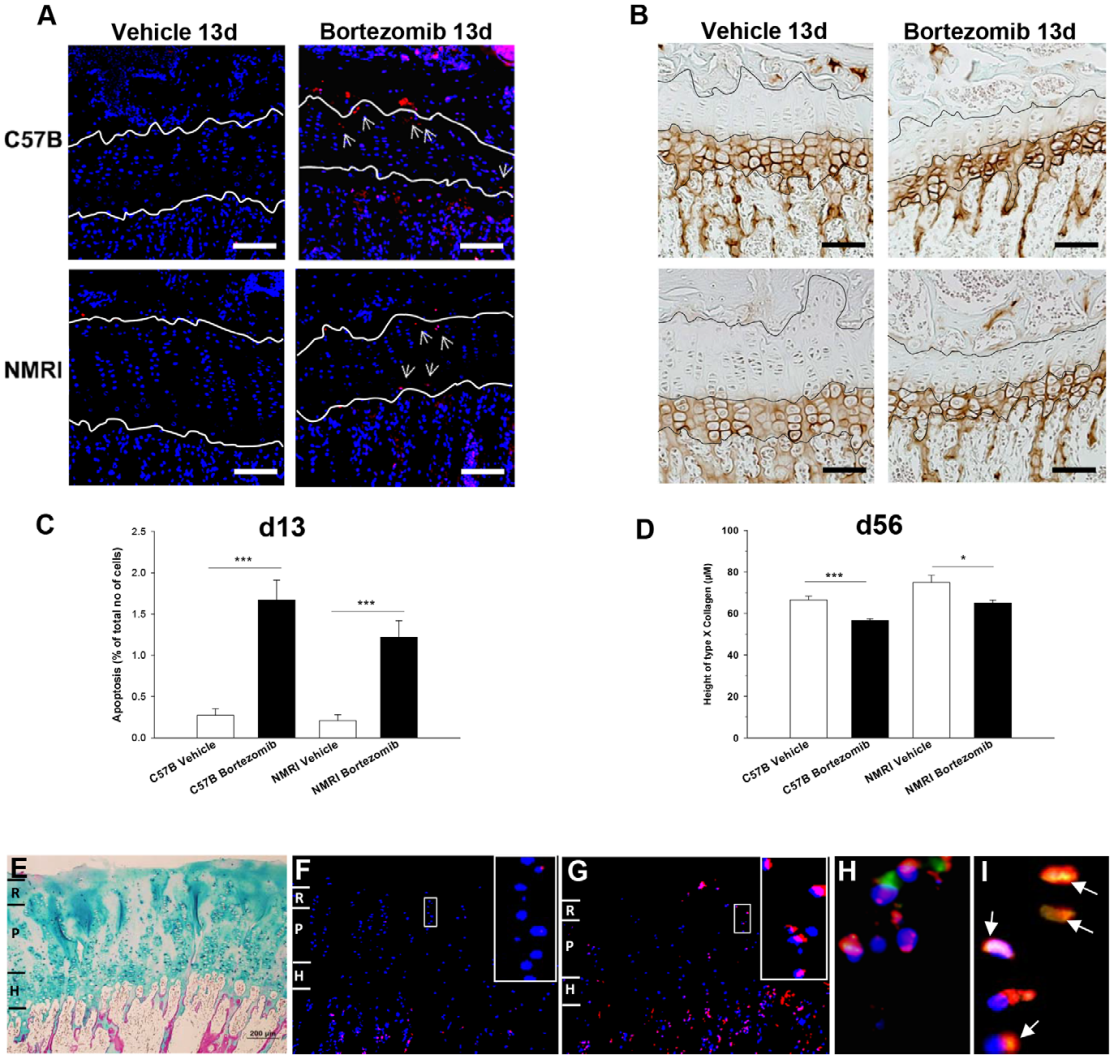
Proteasome inhibition affects chondrogenesis and triggers chondrocyte apoptosis. **A)** Representative micrographs of apoptosis in tibia growth plate cartilage on day 13 in vehicle- and bortezomib treated C57B and NMRI mice. Arrows point to apoptotic cells [58] and blue color represents nucleus (Dapi). **C)** Quantitative TUNEL analyses and the percentage of positive cells in relation to total cell number were calculated for each bone. Representative type×collagen stained sections (**B**) with histological quantification (**D**) from the two mouse strains**.** Bars indicate 100 µm. Data represent mean±SEM; *p<0.05, ***p<0.001 vs. corresponding vehicle treated mouse. **E)** Human growth plate morphology after 24 hrs of culture. Resting (R), proliferative (P) and hypertrophic (H) zone. TUNEL-staining in sliced human growth plate cartilage cultured for 24 hrs in control (**F**) or bortezomib-containing (1000 nM) (**G**) media. Blue color indicates nucleus (Dapi) and red, TUNEL-positive cells (Alexa Fluor 546). **H, I)** Representative merged immunohistochemically stained human growth plate images of Bax (green color), DAPI (blue color, represents nucleus) and Hsp60 (red color, indicates mitochondria) after 24 hrs culture in control (**N**) or bortezomib-containing (1000 nM) (**O**) media. Orange color indicates where activated Bax has translocated to the mitochondria (arrows in **O**). Scale bar indicates 200 µM.

To investigate the underlying mechanisms behind bortezomib-induced bone growth impairment, quantitative histological analyses of growth plates obtained from *in vivo* exposed mice were performed. Growth plate height was found to be significantly decreased in bortezomib treated mice, both at day 13 (p<0.001 compared to vehicle group of each strain; Fig. 4A and C), and day 56 (p<0.01 compared to vehicle group of each strain; Fig. 4B and D). Bortezomib significantly increased apoptosis of resting/stem-like chondrocytes (p<0.001, Fig. 5A and C) and decreased hypertrophic differentiation, as determined by type×collagen staining (Fig. 5B and D). Furthermore, bortezomib treatment significantly decreased total number of growth plate columns (p<0.05), number of hypertrophic chondrocytes per column (p<0.05), terminal hypertrophic chondrocyte size (p<0.05), as well as proliferative zone height (p<0.001) (Table I). However, there was no difference in chondrocyte proliferation, analyzed by BrdU immunohistochemistry after bortezomib treatment (data not shown), not either in the total number of proliferative chondrocytes per column (Table I). Moreover, a similar finding of increased apoptosis after bortezomib treatment was also found in whole growth plate sections of 12-days cultured fetal rat metatarsal bones (perichondrium excluded). For all bortezomib concentration tested (1–1000 nM), a significant dose-dependent increase in chondrocyte apoptosis was observed (Fig. 3B; p<0.001 vs. control bones). Interestingly, apoptosis was most prominent in resting/stem-like chondrocytes, which also explains why metatarsal bones treated for only 24 hrs showed growth arrest. Metatarsal bones were also stained with Alcian Blue/van Gieson (AB/vG) to detect changes of matrix components such as glycosaminoglycans (GAGs, blue color) and collagens (mainly collagen type II, pink color). Indeed, bortezomib decreased the levels of matrix components, as indicated by low levels of GAG-staining (Fig. 3D, bortezomib 1000 nM shown) compared to control bones (Fig. 3C).

**Table 1:** Histomorphometrical analyses in growth plates of C57B and NMRI treated mice.

Day 13	C57BVehicle		C57BBtz	NMRIVehicle	NMRIBtz
**Growth plate height (µm)**		111.6±3.7	95.9±3.3^c^	144.1±5.7	114.3±7.0^c^
**Height of prolif. zone (µm)**		66.9±3.0	52.7±0.9^c^	78.7±3.9	53.9±2.7^c^
**Prolif. chondrocytes (cells/column)**		8.1±0.3	7.2±0.2	8.3±0.3	7.7±0.1
**Hyp. chondrocytes (cells/column)**		4.9±0.2	4.2±0.2^a^	5.4±0.2	5.4±0.4
**Ratio of prolif./hyp. Cells**		1.7±0.1	1.6±0.1	1.6±0.0	1.2±0.1^a^
**Size of terminal hyp. chondrocyte (µm)**		11.5±0.5	9.4±0.7^a^	11.8±0.5	9.7±0.6^a^
**No. of columns/growth plate**		52.0±1.2	44.8±4.9	57.7±4.9	40.4±0.7^a^

Analyses were performed in tibia growth plates collected on day 13 (48 hrs post-treatment) in C57B and NMRI mice. Btz = bortezomib; Prolif. = Proliferative chondrocytes; Hyp = Hypertrophic chondrocytes. Data represents mean±SEM, n = 5–6 per group.

a p≤0.05;

b p≤0.01;

c p≤0.001 compared to vehicle treated animals of the same strain.

### Bortezomib Activates Bax and Induces Apoptosis in Cultured Human Growth Plate Cartilage

Human growth plate cartilage was cultured for 24 hrs in the presence of 1000 nM bortezomib, processed and then stained with AB/vG for morphological studies (Fig. 5E) or analyzed for apoptosis using the TUNEL assay (Fig. 5F and G). Bortezomib was found to significantly increase chondrocyte apoptosis (24.7±4.7% TUNEL positive cells vs. 7.3±1.9% in control; p<0.001), an effect mainly observed in resting/stem-like and early proliferative chondrocytes. In addition, the pro-apoptotic protein, Bax, translocated to the mitochondria in bortezomib treated human growth plate cartilage (Fig. 5I, orange color, arrows), but not in control tissue (Fig. 5H, green color). These results are consistent with the apoptosis data in treated mice and in cultured metatarsals presented above.

### Bortezomib Targets Mainly Resting/Stem-like Chondrocytes

**Figure 6:**
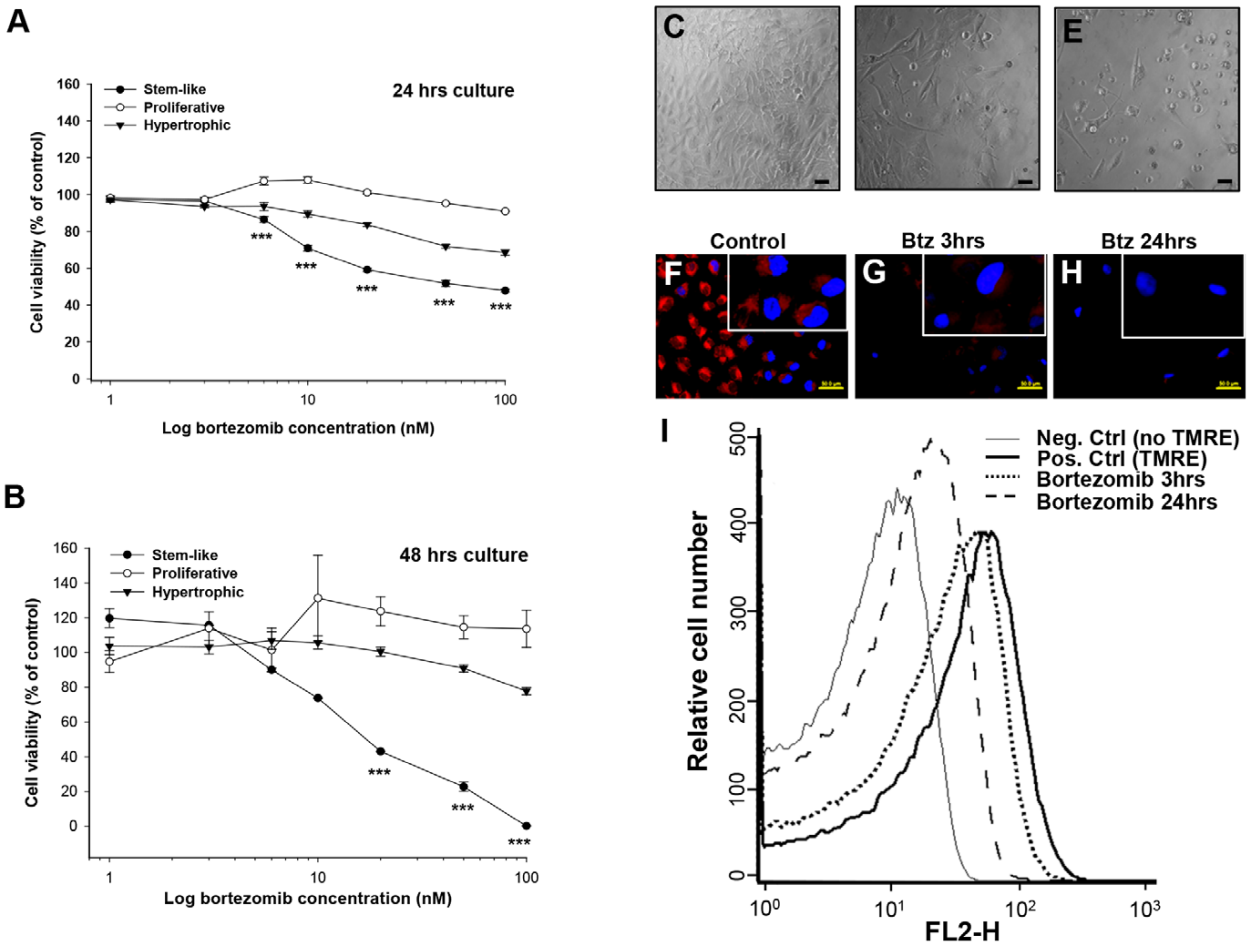
Time- and dose-dependent effects of bortezomib in the rat C5.18 cell-line. C5–18 cells in different phases of chondrogenesis; resting/stem-like (filled circles), proliferative (open circles) and hypertrophic (triangles) were treated with bortezomib (0–100 nM) for 24 (**A**) or 48 hrs (**B**) and assessed for viability. Mean±SEM from 3 individual experiments; ***p<0.001 for resting/stem-like vs. proliferative and hypertrophic chondrocytes. Morphology of resting/stem-like chondrocytes cultured for 24 hrs with control medium (**C**)**,** 100 nM bortezomib (**D**) or 1000 nM (**E**), bars indicate 200 µm. **F–I)** Bortezomib treatment led to loss of mitochondrial membrane potential (ΔΨm) in rat C5.18 resting/stem-like chondrocytes. Representative merged images from TMRE (red color, mitochondria) and DAPI (blue color, represents nucleus) in control (**F**)**,** with polarized mitochondrial inner membranes. Cells treated with 1000 nM bortezomib (Btz) for 3 hrs (**G**) or 24 hrs (**H**) exhibit depolarized mitochondria with reduced red fluorescence. Bars represent 50 µm. Chondrocytes were also analyzed by FACS and treated with either negative control (no addition of TMRE, thin continuous line), positive control (addition of TMRE, thick continuous line), or 1000 nM bortezomib for 3 (dotted line) or 24 hrs (dashed line) (**I**). Apoptotic cells exhibit less TMRE-fluorescence compared to healthy control cells.

Our *in vivo* and *in vitro* data where we observe permanent bone growth impairment suggest that bortezomib targets a specific cell population within the growth plate. To identify whether chondrocyte sensitivity was affected by the stage of differentiation, we used the rat C5.18 chondrogenic cell line which undergo all three stages of chondrogenesis [23], [24]. The cells were treated for 24 or 48 hrs with bortezomib (0–100 nM) and viability was assessed using the MTT-assay. Bortezomib decreased cell viability in a dose and time-dependent manner (Fig. 6A and B). The resting/stem-like chondrocyte population was the most sensitive with an IC_50_ value of 50 nM and 18 nM at 24 and 48 hrs, respectively (Fig. 6A and B, filled circles). In contrast, C5.18 chondrogenic cells representing proliferative and hypertrophic stages of differentiation were more resistant to bortezomib treatment (Fig. 6A and B, open circles and triangles, respectively). When resting/stem-like chondrocytes were studied at earlier time-points, bortezomib (100 nM) was found to significantly increase DNA fragmentation (using cell death ELISA) after 12 hrs (3.8±0.8 fold increase above control; p<0.001), but not at 6 hrs exposure (data not shown). Along with confirming that bortezomib induces DNA fragmentation in a time-dependent manner, we also found that C5.18 resting/stem-like chondrocytes were more sensitive to bortezomib than proliferative chondrocytes (data not shown). Morphological analyses of C5.18 resting/stem-like chondrocytes revealed decreased number of viable cells and significantly increased levels of dead (floating) cells after bortezomib exposure (100 nM and 1000 nM) as compared to the control (Fig. 6D–E vs. C, respectively). Furthermore, cell cycle analyses of bortezomib-treated C5.18 resting/stem-like chondrocytes exhibited a prominent peak in the sub-G_1_ region with a decreased G_1_-peak compared to control (data not shown), indicative of the induction of apoptosis.

### Bortezomib Induces Mitochondrial Membrane Dysfunction in Chondrocytes

Mitochondria play a key role in cell proliferation and viability. Therefore, we next examined the mitochondrial membrane potential (ΔΨm) in C5.18 chondrocytes exposed to bortezomib (Btz). As demonstrated in the merged immunofluorescent images (Fig. 6F–H) of mitochondria (TMRE, red color) and nuclei (DAPI, blue color), bortezomib caused depolarization of the mitochondrial membrane leading to leakage of the lipophilic fluorescent dye, TMRE, which was observed after 3 hrs (Fig. 6G), and moreover at 24 hrs (Fig. 6H). The shift in fluorescence was quantified in a FACSCalibur flow cytometer by overlaying the histograms of the normal (with and without TMRE) and the apoptosis-induced cell population (3 and 24 hrs after bortezomib treatment, Fig. 6I). These results further support a cytotoxic profile of bortezomib in treated chondrocytes.

### Proteasome Inhibition Stabilizes p53 in Chondrocytes

**Figure 7:**
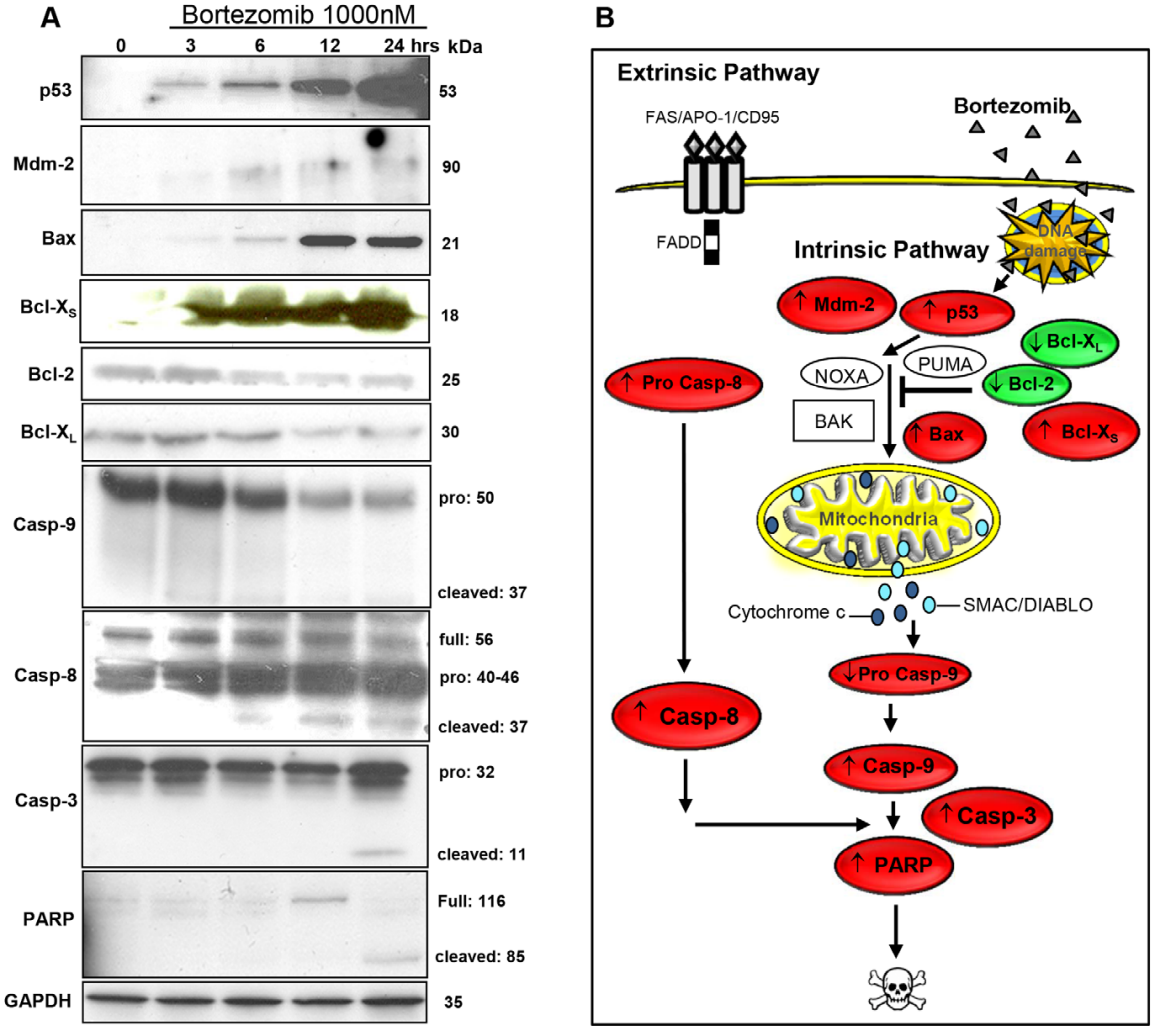
Changes in protein expression induced by bortezomib in resting/stem-like chondrocytes. C5.18 cells were treated with bortezomib (1000 nM) for 3, 6, 12 or 24 hrs. Total cell lysates were assayed by western immunoblotting to detect any changes in pro- and anti-apoptotic proteins. **A)** Bortezomib up-regulated the expression of p53, Mdm-2 and pro-apoptotic proteins Bax and Bcl-X_S,_ as well as induced the cleavage of caspase-9, -8 and -3. The hallmark protein in the apoptotic cascade, PARP was found to be cleaved in bortezomib treated cells. In contrast, the anti-apoptotic proteins Bcl-2 and Bcl-X_L_ were down-regulated. GAPDH is shown as a loading control. **B)** The simplified drawing illustrates how bortezomib may influence several proteins that regulate apoptosis in chondrocytes. Pro-apoptotic proteins are marked in red and anti-apoptotic proteins in green. The arrows indicate if the proteins are up- or down regulated.

Proteasome inhibition is known to cause accumulation and stabilization of different pro-apoptotic proteins normally degraded by the proteasome, including p53 [15]. So far, any role of p53 in bortezomib treated chondrocytes is not known. To address this, protein expression profiles (using Western immunoblot) were determined in resting/stem-like C5.18 chondrocytes exposed to bortezomib (1000 nM) for 3, 6, 12 or 24 hrs (Fig. 7A). We then found that bortezomib effectively caused a pronounced accumulation of ubiquitinated proteins (Fig. S1), as well as p53 (Fig. 7A). Also cultured metatarsal bones treated with bortezomib (1000 nM) showed increased expression of p53 (Fig. 3F), which was not found in control metatarsals (Fig. 3E). p53 has previously been demonstrated to stimulate the expression of several genes, including Mdm-2, a negative regulator of p53 [25]. Indeed, we confirmed that in parallel with early up-regulating p53, bortezomib also induced an accumulation of the Mdm-2 protein (Fig. 7A). Altogether, our data suggest that proteasome inhibition not only stabilizes p53, but also causes transactivation of the p53 target gene, Mdm-2.

### Levels of Bcl-2 Family Member Proteins are Altered by Bortezomib

To further dissect the mechanisms of bortezomib-induced chondrocyte apoptosis, the expression pattern of several members of the Bcl-2 family of proteins was analyzed in resting/stem-like C5.18 chondrocytes. Bortezomib was then found to induce an early (i.e. after 3 hrs) and time-dependent up-regulation of Bax and Bcl-X_S_ (Fig. 7A), two pro-apoptotic members of the Bcl-2 family. In contrast, levels of the anti-apoptotic proteins Bcl-2 and Bcl-X_L_ were slightly decreased at 6 and 12 hrs, respectively (Fig. 7A). These data suggest that Bax plays a crucial role in triggering mitochondrial-mediated apoptosis as it was up-regulated already 3 hrs after exposure to bortezomib.

### Bortezomib Activates the Caspase Cascade

To investigate the initiator caspase, we determined the levels of active/cleaved caspases as depicted in Fig. 7A. Caspase-9 was activated first (at 3 hrs, cleaved 35 kDa fragment), followed by caspase-8 (6 hrs, cleaved 37 kDa fragment), and finally effector caspase-3 (24 hrs, cleaved 11 kDa fragment). Furthermore, we also observed activation of caspase-3 in bortezomib-treated metatarsal bones (Fig. 3H), but not in control metatarsals (Fig. 3G). Cleavage of the nuclear 116 kDa caspase substrate, PARP, to its characteristic 85 kDa fragment is also considered a marker of apoptosis. Herein, we demonstrated that full length PARP was up-regulated at 12 hrs, then proteolysed into its smaller fragment after 24 hrs of bortezomib treatment (Fig. 7A), confirming the enzymatic activation of caspases. Overall, our data support a role for the caspase cascade as a mediator of bortezomib-induced apoptosis in chondrocytes. The cartoon in Fig. 7B illustrates the underlying molecular mechanisms in bortezomib-induced chondrocyte apoptosis.

### Bone Remodeling is not Affected by Bortezomib Treatment

To investigate if bortezomib had any impact on the phenotypic and mechanical characteristics of the long bones, we investigated bone mineral density (BMD), cortical and trabecular parameters as well as bone strength in C57B and NMRI mice treated with bortezomib or vehicle. Our results indicate that none of the tested parameters were significantly affected by bortezomib (Table II and III, respectively), indicating that bortezomib does not have an effect on bone remodeling. Moreover, biomarkers of bone formation (type I procollagen N-terminal, PINP) and bone degradation (collagen type 1 cross-linked C-telopeptide, Ctx) do not either show any significant differences between the treatment groups of the same strain (PINP = 95.6±11.8 ng/ml in C57B vehicle vs. 107.9±18.4 ng/ml in bortezomib and 127.4±13.6 ng/ml in NMRI vehicle vs. 156.5±26.2 ng/ml in bortezomib, and Ctx = 34.5±1.5 ng/ml in C57B vehicle vs. 39.6±4.7 ng/ml in bortezomib and 39.2±1.6 ng/ml in NMRI vehicle vs. 42.7±2.8 ng/ml in bortezomib).

**Table 2:** pQCT analyses in C57B and NMRI treated mice on day 13 and 56.

Day 13	Total BMD	Trab. BMD	CRT_CNT	CRT_DEN	CRT_A
	(mg/cm^3^)	(mg/cm^3^)	(mg/mm)	(mg/cm^3^)	(mm^2^)
**C57B Vehicle**	365.3±7.3	245.7±6.4	0.7±0.02	1010.7±4.8	0.6±0.02
**C57B Btz**	389.8±14.9	252.4±19.9	0.7±0.02	1010.8±4.1	0.7±0.02
**NMRI Vehicle**	471.1±11.6	286.8±12.2	1.0±0.05	1139.9±14.9	0.9±0.03
**NMRI Btz**	476.8±20.1	315.3±16.1	0.9±0.05	1127.6±4.5	0.8±0.04
**Day 56**					
**C57B Vehicle**	377.8±7.2	187.1±3.7	0.9±0.04	1115.5±8.8	0.8±0.03
**C57B Btz**	391.6±8.2	197.4±7.0	0.8±0.03	1098.2±8.4	0.8±0.02
**NMRI Vehicle**	470.1±21.4	194.5±20.2	1.4±0.1	1261.9±9.1	1.1±0.04
**NMRI Btz**	476.9±19.8	213.2±18.4	1.4±0.1	1255.3±19.1	1.1±0.05

pQCT was used to determine the trabecular BMD in the metaphyseal region of the left tibia and cortical area, thickness, and BMD in the diaphyseal region of the left tibia in C57B and NMRI treated mice on day 13 (48 hrs post-treatment) and on day 56 (45 days post-treatment). Btz = bortezomib, Trab BMD – trabecular bone density, CRT_CNT – cortical content, CRT_DEN – cortical density, CRT_A – cortical area. Values indicate mean±SEM, n = 5 per group.

**Table 3:** Biomechanical testing of bone strength in C57B and NMRI treated mice.

Day 13	Breaking Force	Structural Stiffness
	(N)	(N/mm)
**C57B Vehicle**	11.5±0.6	43.2±6.9
**C57B Btz**	11.4±0.4	45.3±5.4
**NMRI Vehicle**	18.9±0.3	77.8±5.7
**NMRI Btz**	17.7±1.1	68.9±6.0
**Day 56**		
**C57B Vehicle**	14.4±0.6	72.1±6.2
**C57B Btz**	14.0±0.7	71.1±3.8
**NMRI Vehicle**	23.0±0.8	111.9±7.1
**NMRI Btz**	20.9±0.7	107.7±5.1

Biomechanical testing of bone strength was performed by 3-point bending analyses in C57B and NMRI treated mice. Analyses was performed on day 13 (48 hrs post-treatment) and on day 56 (45 days post-treatment). Btz - bortezomib, Breaking force (N) – the maximum load the bone can take before breakage, Structural stiffness (N/mm). Values indicate mean±SEM, n = 7–9 per group.

## Discussion

Herein, we show that systemic administration of a clinically relevant dose of bortezomib targets the growth plate and permanently impairs linear bone growth in young mice, an effect linked to increased apoptosis and decreased differentiation/hypertrophy of growth plate chondrocytes. This effect was mediated through a local action of bortezomib in the growth plate, causing permanent growth failure and increased resting/stem-like chondrocyte apoptosis. We also show that bortezomib mainly acts via the classical intrinsic apoptotic pathway, in which p53 and Bax appear to be the key regulators triggering apoptosis. In addition we confirmed that also cultured human growth plate cartilage is highly sensitive to bortezomib, which exerts cytotoxic effects mainly by triggering apoptosis in resting/stem-like chondrocytes.

In three different *in vivo* mouse studies, including two strains of mice and animals either fed *ad libitum* or pair-fed, we consistently found that bortezomib caused severe growth impairment, an effect which was persistent when following the animals for up to 6 months after treatment. The observed effect was linked to increased apoptosis of resting/stem-like chondrocytes as well as decreased differentiation/hypertrophy. These observations are in line with our previous report where the non-clinically used proteasome inhibitor, MG262 impaired bone growth in treated mice [17], [18].

To investigate if bortezomib may act systemically to modulate the GH/IGF-I system to impair bone growth, serum IGF-I levels were measured. As no suppression of serum IGF-I was found, our data supports a more local action of bortezomib, directly targeting the growth plate chondrocytes. This was further confirmed in organ cultures of fetal and postnatal rat metatarsal bones, were bortezomib efficiently caused permanent growth arrest. Indeed, even a short, 24 hrs exposure to bortezomib was sufficient to cause permanent growth arrest of cultured fetal rat metatarsal bones. Using cultured human growth plate cartilage, we were able to reproduce our *in vivo* and metatarsal data showing a direct cytotoxic effect of mainly resting/stem-like chondrocytes. Therefore, our findings strongly support the hypothesis that bortezomib has a local harmful effect on growth plate chondrocytes and thereby could affect linear bone growth in treated children. Nevertheless, we cannot exclude that systemic factors other than IGF-I may play a role in bortezomib induced growth impairment.

Our results in mice, rat and human growth plate cartilage provide evidence that resting/stem-like chondrocytes are the main target of bortezomib, thereby further explaining an incomplete catch-up bone growth as observed in treated mice as well as in cultured metatarsal bones. Previous reports show that resting/stem-like chondrocytes acts like a limited pool of cells, with only a limited number of cell divisions, feeding daughter cells into the adjacent proliferative cell layer and makes important contribution to endochondral bone formation [26], [27]. The fact that resting/stem-like chondrocytes were indeed the main target, and that not a complete catch-up growth occurred after bortezomib treatment supports the importance of the resting zone chondrocytes, and that loss of them may lead to loss of growth potential. These observations are in agreement with our previous study, were MG262 caused impaired bone growth and significant induction of resting/stem-like chondrocyte apoptosis, both *in vitro* and *in vivo* [17], [18]. Our findings that bone growth is negatively influenced by proteasome inhibitors is further supported by a recent report where they show that proteasome inhibitor I (PSI) reduced metatarsal bone growth and induced chondrocyte apoptosis, mediated mainly by an effect on proliferative and hypertrophic chondrocytes [19]. In contrast, our data are reproduced both *in vivo* and *in vitro*, including human growth plate cartilage and consistently show that resting/stem-like cells are the main target, and as a consequence, causes severe growth failure. It should be emphasized that PSI is not in clinical use and belongs to the peptide aldehyde class of proteasome inhibitors, which are less specific and potent [28]. In comparison, both MG262 and bortezomib belongs to the peptide boronic acid class of proteasome inhibitors in which bortezomib is approved for treatment of multiple myeloma and mantle cell lymphoma in adults [12], [13] and are under clinical trials in children with cancers [20], [21]. Altogether, it appears that inhibition of proteasome function in chondrocytes specifically induces apoptosis, linking the UPS of protein degradation with the regulation of apoptotic cell death in chondrocytes and, in turn, with negative consequences on longitudinal bone growth.

Induction of apoptosis by bortezomib implies that chondrocyte death is induced as a result of increased intracellular concentrations of a regulatory molecule(s) normally degraded by the UPS. A prime candidate for such a regulator of apoptosis appeared to be the transcription factor, p53, which is known to be ubiquitinated and degraded by the proteasome [29]. We found that treatment with bortezomib indeed resulted in stabilization and rapid accumulation of p53, shown to be biological active, based on transactivation of the p53 target gene encoding Mdm-2. Current evidence indicates that the mode of action of p53-mediated apoptosis involves transactivation of target genes, such as the Bcl-2 family of proteins [29]. Present data indicate that bortezomib could also down-regulate the anti-apoptotic Bcl-2 protein in chondrocytes. The pro-apoptotic Bcl-2 family member, Bax, forms heterodimers with Bcl-2 and causes accelerated cell death when overexpressed. In agreement with this, bortezomib treatment was found to significantly increase the expression of both Bax and Bcl-X_S_ and decrease levels of the anti-apoptotic protein, Bcl-X_L_. Bax has been demonstrated to impair mitochondrial membrane stability and promote apoptosis [30]. Indeed, in our model system we found that in parallel to transactivating Bax, bortezomib also decreased mitochondria membrane potential which has been implicated as a key mechanism involved in apoptosis in various cell death paradigms [31].

In chondrocytes, we found that the outer mitochondrial membrane becomes permeable, leading to activation and cleavage of pro-caspase-9, followed by activation of the executioner caspase-3, with subsequent cleavage of PARP. In multiple myeloma cells, bortezomib has been shown to activate both the intrinsic apoptotic pathway, involving mitochondrial cytochrome c release and caspase-9, as well as the extrinsic, Fas/caspase-8-dependent apoptotic pathway [14]. We also noticed activation and cleavage of caspase-8, without activation of the death receptor, Fas (data not shown). It is not clear how bortezomib can activate caspase-8, which is normally activated through death receptor activation (extrinsic). However, one could speculate that bortezomib may induce caspase-8 activation through activated caspase-9, as confirmed in other cell types [32], [33], [34], [35]. In addition, it has also been demonstrated that activated caspase-3 can activate caspase-8 in MCF7 cells [36]. Furthermore, p53 can directly engage each of the major apoptotic pathways, stimulating both death receptor signaling and the intrinsic mitochondrial pathway, including cytochrome c release [15]. Thus, even though a hierarchy of caspase activation has been established in many circumstances, these results suggest that this hierarchy may not be absolute and that significant cross-talk between the death receptor and mitochondrial-signaling pathways can occur. Our findings suggest that the intrinsic apoptotic pathway, involving early activation of p53 and Bax, followed by the downstream events of caspase cleavage, might be the dominant mechanism for bortezomib-induced apoptosis in chondrocytes. Thus, small molecule based therapy targeting p53 [37] and/or Bax [38] regulatory proteins may be one possible way to prevent from eventual undesired secondary effects in normal tissues after bortezomib treatment, without interfering with the desired anti-cancer effect.

A consequence of new and more intensive chemotherapy regimens in children may lead to a reduced adult height, and decreased bone mineral density leading to osteopenia, osteoporosis, and pathological fractures later in life [39], [40], [41], [42]. Interestingly, we found no significant effect of bortezomib on BMD, serum bone biomarkers (PINP and Ctx) or bone biomechanical properties, such as cortical content, cortical thickness or bone strength. Previous studies have shown that proteasome inhibitors such as proteasome inhibitor-1, epoxomicin and bortezomib may enhance bone formation and BMD in 5-weeks-old Swiss ICR white mice [43] and in 7-week-old C57B/6 mice [44]. However, in another study, bortezomib had no effect on femur BMD in a myeloma model of 15-week-old CB.17/Icr-SCID mice [45], which is in line with our data. These conflicting results may suggest that regulation of mouse bone remodeling by the UPS is influenced by age, mouse strain, dose, duration of treatment and/or immune function. The impact of proteasome inhibitors on different bone parameters in children is still unknown.

Worldwide, the most common cause of growth retardation is malnutrition [46], which clearly indicate the importance of the nutritional status for bone- and growth plate microenvironment and thereby normal linear bone growth. In mice fed *ad lib*, a significant decrease in body weight was observed during treatment with bortezomib compared to vehicle. Comparable observations are also commonly seen in treated cancer patients, showing decreased appetite, well-being and body weight. However, it was important to verify if bortezomib by itself had a growth inhibiting effect, and that the observed effect was not because of different nutritional status, thereby we conducted a pair-feeding experiment. The pair-fed experiment was additionally performed in two strains of mice to conclude that there was not a strain-specific effect. A slight decrease in body weight in the NMRI strain of mice was observed on day 36 until termination on day 56 when challenged to bortezomib. However, differences in body weight response to drugs have previously been reported between C57B and NMRI mice [47], and could be linked to the fact that C57B is an inbred stain whereas NMRI is an outbred strain of mice. The dose used, (1 mg/kg, corresponding to 3.0 mg/m^2^) has been shown to be the maximum tolerated dose producing greatest antitumor activity in different human xenograft models [48], is more than twice the dose used in humans (1.3 mg/m^2^). However, the basal metabolic rate per gram of body weight is seven times higher in mice than in humans [49]. In addition, the measurement of proteasome inhibition is the clinical marker for a targeted effective dose and it should be in the 50–80% range [22], which we also demonstrated in our study, indicating a clinically relevant dose. The highest *in vitro* bortezomib dose used (1000 nM) is achievable in plasma of multiple myeloma patients, were median plasma bortezomib concentration was 509 ng/mL (range: 109–1300 ng/mL, corresponding to 1320 nM) (Product information. Velcade (bortezomib). Cambridge, MA: Millenium Pharmaceuticals, March 2006). It is important to remember that humans and rodents (mice) are different with regard to linear bone growth, which is the main limitation of this study. Humans show a prominent growth spurt at time of puberty, which is not the case in mice. Moreover, humans fuse their growth plates at end of puberty, while mice growth plates stay open their whole life. However, we validated our results also in cultured human growth plates from pre-pubertal children and found similar results as in rodent growth plate chondrocytes, strengthening our observations. Furthermore, all mammalian cells use similar molecular mechanisms to regulate growth, replication, differentiation and death [49], further supporting our observations. It should also be noted that the mice were followed up to 6 months after last injection, corresponding to adulthood in a mouse, and the animals were still growth retarded. However, based on our study it is not possible to conclude what would happen to the growth beyond these 6 months.

Altogether, we provide evidence that a clinically relevant dose of bortezomib exerts cytotoxic effects mainly on resting/stem-like growth plate chondrocytes, resulting in permanent impairment of linear bone growth. Our confirmation that human growth plate chondrocytes are highly sensitive to bortezomib-induced cytotoxicity stresses the importance of long-term follow up of children treated with proteasome inhibitors to determine to what extent longitudinal bone growth may be impaired.

## Materials and Methods

### Ethics Statement

The human growth plate tissue collection protocol was approved by the local medical ethics committee (Karolinska Institutet Research Ethics Committee North at the Karolinska Hospital, Stockholm, Sweden). According to this approval, verbal informed consent was obtained from all patients and their legal guardians and documented in the original hospital records. All clinical investigation was conducted according to the Helsinki Declaration.

All animal studies were carried out in strict accordance with the regulations issued by Swedish National Board for Laboratory Animals (SFS 1988∶541). The protocol was approved by the local animal ethics committee (Stockholm North Animal Ethics Committee, Permits number: N49/06; N9/07 and N283/07). All efforts were made to minimize animals from suffering.

### Reagents

The proteasome inhibitor bortezomib (Velcade™; formerly known as PS-341, LDP-341 and MLM341, Millennium Pharmaceuticals, Cambridge, MA) was dissolved in sterile saline (0.9%) to a final concentration of 1 mg/ml or 2.6 mM, aliquoted and stored at −80°C. Modified Eagle’s Medium (MEM), Modified Eagle’s Medium (MEM) alpha and Dulbecco’s Modified Eagle’s Medium (DMEM)-high glucose containing 25 nM HEPES, L-Glutamine and 4.5 g LD-glucose, fetal bovine serum (FBS), fungizone, gentamicin, sodium pyruvate, penicillin/streptomycin, PBS, EDTA and Trypsin-EDTA solutions were all purchased from Invitrogen Inc. (Paisley, UK). Bovine serum albumin (BSA), β-glycerophosphate, ascorbic acid and dexamethasone were all purchased from Sigma-Aldrich (Schnelldorf, Germany). Mounting medium containing DAPI was purchased from Vector Laboratories (Burlingame, CA).

### Animals

Two strains (C57B and NMRI, n = 8–10/group) of 5-week old intact male mice (Scanbur Nova-SCB, Sollentuna, Sweden), one per cage, were housed in our animal care facility. After an adaptation period, each strain of mice were randomized into two groups based on their body weight and then weight-matched into pairs to receive either 1 cycle of intraperitoneally (i.p.) injected bortezomib (1 mg/kg on day 1, 4, 8 and 11) or vehicle (sterile saline) (Fig. 2A). Body weight, food intake and general physical status was recorded daily during the first 20 days of treatment, and each vehicle-treated mouse was pair-fed (standard pellet) to the corresponding weight-matched bortezomib treated animal. Femur bone growth was followed longitudinally by X-ray (GE AMX-4, GE Healthcare, USA, with the settings: 50 kV, 2.5 mAs) analyses under light isoflurane anesthesia, before start of treatment (at day 0), at time of sacrifice (day 13, day 56 or 6 months; Fig. 2A) and approximately every second week in-between. In another set of experiment, C57B mice (n = 10 mice per group) were fed *ad libitum*, randomized into two groups and housed 5 per cage to either receive 1 cycle of i.p. injected bortezomib (1 mg/kg on day 1, 3, 6 and 8) or vehicle (sterile saline; Fig. 1A). In that study, bone growth was followed by dual X-ray absoptiometry (DXA, Norland pDEXA Sabre and the Sabre research software (Version 3.6; Norland Medical Systems, Fort Atkinson, WI) under light isoflurane anesthesia, before start of treatment (at day 0), at time of sacrifice (day 10 or day 53) and approximately every second week in-between (Fig. 1A). To study cell proliferation, bromodeoxyuridine 5-bromo-2-deoxyuridine (BrdU, RPN 20; Amersham Biosciences, Buckinghamshire, UK) was injected twice (at 16 and 2 hrs) before being euthanized by an overdose of CO_2_. In both of the above mentioned experiments, three out of the total 65 vehicle treated mice died (4.6%) and 6 of the total 66 bortezomib treated (9.1%) died early during the treatment period and were excluded from the experiments and further analyses. Tibias and femurs were dissected out and fixed in 4% formaldehyde for 24 hrs at 4°C. After fixation, specimens were decalcified in 10% EDTA and then paraffin embedded. Serum levels of insulin-like growth factor-I (IGF-I) was measured on day 13 and 56 using a commercial radioimmunoassay kit (Mediadiagnostic, Tubingen, Germany) according to the manufacturer instructions. Quantitative histomorphometrical evaluations in Alcian Blue/van Gieson-stained tibia growth plate sections were performed as previously described [50] by a person blinded to the experimental details.

### Organ Cultures

The three middle metatarsal bone rudiments were dissected out from the hind paws of 20-days-old rat embryos (E20) or 8 days (P8) old rats and cultured as previously described [51], [52]. Images were captured at days 0, 2, 5, 7, 9 and 12 of culture using a Hamamatsu C4742–95 digital camera mounted on a Nikon SMZ-U microscope, and bone lengths were measured and quantified as previously described [51]. Treatments with various concentrations of bortezomib began on day 0, and the medium was changed every 2–3 days. Each experiment was repeated 2–3 times (5 to 6 bones per group).

### Cell Line and Culture Conditions

The RCJ3.1C5.18 (C5.18) rat chondrogenic cell line [53] was a kind gift of Dr. Anna Spagnoli, used and cultured as previously described [23], [24]. Briefly, after reaching confluence (resting phase; 4 days), cells were treated with fresh MEM alpha supplemented with 50 µg/ml ascorbic acid and 10 mM β-glycerophosphate. After 4–7 days of culture, the cells acquire markers of early chondrocytic differentiation (type II collagen and proteoglycan synthesis, proliferative phase) and progressively acquire markers of terminal differentiation (type×collagen and alkaline phosphatase activity, hypertrophy) at 10–14 days of culture. The cultures were monitored over a total period of 12 days and cultures were supplemented with fresh MEM alpha every 3 days.

### Human Growth Plate Cultures

Growth plate biopsies were collected from the proximal tibia and distal femur of 6 pubertal children (3 boys and 3 girls) undergoing epiphyseal surgery for different medical conditions (4 patients with constitutional tall stature, 1 with leg length discrepancy, and 1 with androgen insensitivity). Epiphyseal biopsies were collected with a special biopsy needle (Gallini Biomid, size 7G 10 cm, Apgar, Denmark), transferred to tubes containing DMEM-high glucose supplemented with 20 µg/ml gentamicin, and placed directly on ice in the operating room. In the lab, biopsies were cut into ½–1 mm thick slices under an inverted microscope (Zeiss Stemi DV4, Stockholm, Sweden), transferred into individual 24-well plates, and cultured for 24 hrs in 1 ml of DMEM-high glucose supplemented with 20 µg/ml gentamicin, 50 µg/ml ascorbic acid, 216 µg/ml beta-glycerophosphate, and 0.2% BSA at +37°C with 5% CO_2_. After the culture period, samples were fixed in 4% formalin for 24 hrs, decalcified in 10% EDTA pH 7.8 for 24 hrs, embedded in paraffin, and cut into 4 µm thick slices. All tissue samples were processed in the same way and at least 3 slices per group from each patient were collected.

### Pharmacodynamics

Proteasome activity was measured in blood after a single i.p. injection of bortezomib (1 mg/kg) or vehicle in C57BL and NMRI mice. Blood was collected in tubes containing Heparin (100IE/KY/ml, LEO Pharma, Malmö, Sweden) before treatment (0 hrs) and 1, 24 and 72 hrs post-treatment (n = 3 mice per time point). Chymotrypsin-like proteasome activity was conducted using the synthetic flourometric substance method [54].

### Analyses of Bone Parameters and Biomarkers

Tomographic pQCT bone measurements were performed using the Stratec XCT Research M (software version 5.4B; Norland Medical Systems) and trabecular and cortical parameters were evaluated as described previously [55]. Mechanical properties of the femoral shafts were tested with the 3-point bending method using a universal mechanical testing device (Avalon Technologies, Rochester, MI, USA). The span length was 7 mm. Each femur was compressed at a constant rate of 10 mm/min until breakdown. Mechanical parameters, including breaking force (maximal load, N) and slope of the load-deformation curve, representing structural stiffness (N/mm) were calculated from the raw data files using Origin software (Origin Lab Corp. Northampton, MA, USA). Serum levels of type 1 procollagen N-terminal (PINP) and collagen type 1 cross-linked C-telopeptide (Ctx) was measured on day 13 using a commercial radioimmunoassay kit (Immunodiadiagnosticsystem, Boldon, United Kingdom) according to the manufacturer instructions. The serum samples from each animal were run in duplicate, n = 7–9 animals per group).

### Cell Viability and Apoptosis

Resting/stem-like chondrocytes C5.18 cells [23], [24] were treated with bortezomib for 24 or 48 hrs in 96-well plates. Cell viability was evaluated using a colorimetric assay (3-(4,5-dimethylthiazol-2-yl)-2,5-diphenyltetrazolium bromide, MTT, Sigma-Aldrich, Schnelldorf, Germany), as previously described [56]. Each experiment included six wells and was repeated three times. Apoptosis was analyzed by the detection and quantification of cytoplasmic histone-associated DNA fragments (mono- and oligonucleosomes), as previously described [57]. Each experiment included two replicates per sample and was repeated three times. For DNA content quantification, cells were harvested, fixed with 70% ethanol, treated with RNase (0.25 mg/ml, BD Pharmingen, MA) and stained with propidium iodide (PI, 0.05 mg/ml, Sigma-Aldrich, Schnelldorf, Germany). Samples were incubated at 37°C for 30 min before being analyzed on a FACSCalibur flow cytometer (Becton Dickinson Immunocytometry Systems, San Jose, CA), according to standard procedures.

### Mitochondrial Membrane Potential (Δψm)

C5.18 chondrocytes were plated into 8-well sterile chamber slides, cultured for 4 days, and treated with 1000 nM bortezomib for 0 to 24 hrs. Cells were then incubated with 0.2 µM tetramethylrhodamine ethyl ester (TMRE, Molecular Probes) for 30 min at 37°C and 5% CO_2_, before being embedded with DAPI mounting medium for subsequent ΔΨm microscopy analyses. For each visual field, three images were captured: a light image, a DAPI image (detection of all cells, emission at 449 nm, blue color) and a TMRE image (ΔΨm-positive cells, emission at 573 nm, red color). For quantification of ΔΨm, samples were analyzed on a FACSCalibur flow cytometer. Unfixed resting/stem-like C5.18 chondrocytes were treated as described above. TMRE-fluorescence was detected in live cells as determined by forward-scatter and side-scatter criteria, and data was analyzed in FLOW JO (version 6.4.7, Ashland, OR). As a negative control, we used only PBS without TMRE.

### SDS-page and Immunoblotting

C5.18 resting/stem-like chondrocytes were treated with 1000 nM of bortezomib for various time-points, and Western blots were performed as described previously [57]. The primary antibodies against p53, Bax, Bcl-X_S/L_, Bcl-2, caspase-8, caspase-3, Poly-ADP-ribose polymerase (PARP), MDM2, ubiquitin (Ub) and GAPDH (1∶1000) were all purchased from Santa Cruz Biotechnology, California. Antibody against caspase-9 (1∶1000) was from Cell Signaling, Massachusetts. After incubation with corresponding horseradish peroxidase conjugated secondary antibodies (Santa Cruz Biotechnology and GE Healthcare, Uppsala, Sweden), bound antibodies were visualized using enhanced chemiluminiscence (ECL^plus^, Thermo Scientific, Rockford), and films were developed.

### Detection of DNA Fragmentation *in situ*

Apoptotic cells were identified by terminal deoxynucleotidyl transferase (TdT)-mediated deoxy-UTP nick end labeling (TUNEL), as previously described [51]. The number of TUNEL-positive chondrocytes was expressed as percent of the total number of cells (DAPI-positive cells) in the same area. For positive and negative controls, we used slides supplied in the kit. For each experimental group, five bones were analyzed in at least two independent experiments.

### Immunohistochemistry

Samples were analyzed with immunohistochemistry as described previously [17]. Primary antibodies for p53 (1∶150, Santa Cruz Biotechnology), active caspase-3 (1∶50, Abcam, Cambridge, Massachusetts), and the secondary biotinylated antibody were purchased from DAKO, Stockholm, Sweden. Bortezomib-treated mouse testes were used as positive- and negative controls. For human growth plates, the primary antibodies, Bax (1∶100; BD Pharmingen, Maryland) and Hsp60 (1∶100; Santa Cruz Biotechnology), were simultaneously incubated in 1% BSA overnight at +4°C, and the fluorescent secondary antibodies, goat anti-mouse IgG2a FITC (1∶300; Santa Cruz Biotechnology) and donkey anti-rabbit Cy3 (1∶300; Jackson Laboratories, Maine), were added for 60 min at room temperature.

### Statistical Analysis

Results are presented as mean values±SEM. Differences between two groups (bortezomib vs. vehicle/control) were evaluated by Student’s *t*-test. Differences between several groups were tested by one-way analysis of variance (ANOVA) followed by the Newman-Keuls post-test. The minimal level of significance was p≤0.05.

## Supporting Information

Figure S1 **Bortezomib cause accumulation of ubiquitinated proteins.** Western blot analysis of whole cell lysates in rat resting/stem-like C5.18 chondrocytes treated with bortezomib (1000 nM) for 3, 6, 12 or 24 hrs, showing accumulation of ubiquitinated proteins.
(TIF)
